# Comparison of different testosterone formulations discontinuation and dose spacing on hematocrit and testosterone levels in transgender individuals with erythrocytosis

**DOI:** 10.61622/rbgo/2025rbgo35

**Published:** 2025-07-15

**Authors:** Sérgio Henrique Pires Okano, Silvio Antônio Franceschini, Luiz Gustavo Oliveira Brito, Lucia Alves da Silva Lara

**Affiliations:** 1 Universidade de São Paulo Faculdade de Medicina de Ribeirão Preto Ribeirão Preto SP Brazil Faculdade de Medicina de Ribeirão Preto, Universidade de São Paulo, Ribeirão Preto, SP, Brazil.; 2 Faculdade de Ciências Médicas de Campinas Campinas SP Brazil Faculdade de Ciências Médicas de Campinas, Campinas, SP, Brazil.

**Keywords:** Transgender persons, Gender-affirming procedures, Testosterone, Hematocrit, Polycythemia

## Abstract

**Objectives:**

This study aimed to compare the effect of Gender Affirming Hormone Therapy with Testosterone (GAHT-T) discontinuation and dose spacing on Hct, hemoglobin (Hb) and total testosterone levels in users of testosterone cypionate and testosterone undecanoate.

**Methods:**

This retrospective cohort analyzed data collected from the medical records of trans men older than 18 years who developed erythrocytosis during GAHT-T between 2020 and 2023. Participants were divided into three groups according to formulation in use when the diagnosis of erythrocytosis occurred and analyzed according to therapeutic approaches: discontinuation of GAHT-T for 3 months or, for the testosterone cypionate fourthly users, dose spacing.

**Results:**

A total of 49 trans men (mean age, 28.0 ± 7.8 years) were diagnosed with erythrocytosis, totalizing 104 tests. After discontinuing GAHT-T, a greater decrease in Hct, Hb and total T levels was observed in the testosterone cypionate (Cip 14 and Cip 28) users than in the testosterone undecanoate users (Und90). In Und90, discontinuation resulted in decrease of Hct and Hb levels, without difference in total T levels. Cip14 and Cip28 exhibited greater reductions in the Hct level than Und90 did with discontinuation. In Cip14, dose spacing had no effect on decreasing Hb, Hct and total T levels.

**Conclusion:**

Discontinuation of testosterone undecanoate for 3 months in trans men undergoing GAHT-T who had developed erythrocytosis reduces hemoglobin and hematocrit levels without a significant reduction in testosterone levels. Dose spacing in fortnightly testosterone cypionate users was not effective to reduce hematocrit and hemoglobin.

## Introduction

Transgender individuals who were assigned female at birth (AFAB) may require gender-affirming hormone therapy with testosterone (GAHT-T) to develop masculine characteristics. This hormone can be administered transdermally or intramuscularly, using either medium-acting esters (testosterone cypionate) or long-acting esters (testosterone undecanoate).^(1)^

During the gender hormone affirmation process, intramuscular administration of testosterone esters is widely used due to its ease of management and cost-effectiveness. Testosterone cypionate has a half-life of 6–8 days and is typically administered intramuscularly every 15 to 21 days. Testosterone undecanoate reaches its peak effect around the first week and is prescribed at approximately 12-week intervals to maintain serum testosterone levels.^(1)^

Although GAHT-T aids trans men in developing masculine traits, it can also influence the lipid profile and elevate systolic blood pressure.^(1-3)^ Additionally, GAHT-T can raise hematocrit (Hct) levels by up to 4.4% (95% confidence interval [95% IC], 3.5–5.3),^(4)^ potentially causing erythrocytosis (Hct≥50%). This condition may result in the necessity of temporary discontinuation of GAHT-T because of the increased risk of cardiovascular events associated with heightened blood viscosity.^(4-6)^

Erythrocytosis in cisgender men can be treated with phlebotomy, antiplatelet drugs, and testosterone (T) dose reduction.^(7)^ Although these treatments can be administered to trans men, no specific therapy or data demonstrating the safety of these practices in trans men presenting with erythrocytosis secondary to T use is available. Thus, the objective of this study was to compare the effect of discontinuing GAHT-T for 3 months on the Hct and total T levels of users of testosterone cypionate and testosterone undecanoate with erythrocytosis, and to compare the effect of fortnightly testosterone cypionate dose spacing and discontinuation in trans men treated in a specialized outpatient clinic.

## Methods

This retrospective cohort study collected data from the medical records of trans men treated at the Gender Incongruence Outpatient Clinic of the *Hospital das Clínicas da Faculdade de Medicina de Ribeirão Preto*, University of São Paulo between 2020 and 2023. This clinic fosters multidisciplinary healthcare for the transgender population, with blood tests and clinical evaluation being conducted on a quarterly basis in the first year of GAHT-T, and then twice a year over the subsequent years of follow-up, in accordance with international guidelines.^(1,8)^

In our service, testosterone cypionate is initially prescribe every 21 days. Patients with lower levels of T on follow-up evaluation have the dose adjusted to 14 days; those who elevate the levels of Hct or present laboratorial increase in transaminases or have T levels over 1200 have the adjustment of the dose to 28 days. Testosterone undecanoate is prescribe to all patients every 3 months (90 days).

In cases of erythrocytosis, the standard therapeutic approach typically involves suspending GAHT-T for three months. However, for patients receiving testosterone cypionate every 14 or 21 days, an alternative strategy may involve extending the dosing interval to 28 days. Treatment adjustments are evaluated based on the patient's clinical condition, considering the potential risks of discontinuation, including bleeding and exacerbation of gender dysphoria. All assessments in this study were conducted three months after the intervention (either discontinuation or dose adjustment).

The inclusion criteria were trans men treated at the Gender Incongruence Outpatient Clinic, over the age of 18 years, who developed erythrocytosis during follow-up using testosterone undecanoate or testosterone cypionate. The exclusion criteria were polycythemia vera or secondary to other pathologies (including chronic kidney disease, severe lung disease, chronic myeloid leukemia, lymphoma, liver disease, or Cushing's syndrome) or loss to longitudinal assessment follow-up. Participants presenting with erythrocytosis on more than one occasion were reincluded in the study.

Participants were categorized into three groups based on the formulation and dosage of testosterone administered at the time of erythrocytosis diagnosis: 1) The "Cip14 group" comprised users of testosterone cypionate 200 mg/2 mL every 14 or 21 days, 2) the "Cip28 group" comprised users of testosterone cypionate 200 mg/2 mL every 28 days, and 3) the "Und90 group" comprised users of testosterone undecanoate 1,000 mg/4 mL every 3 months. The Cip 28 group consisted of patients who required an extension of their testosterone cypionate dosing interval to 28 days for any reason, including those who underwent dose spacing due to erythrocytosis. Comparisons were made both within these groups and in relation to the proposed therapeutic strategies: either discontinuation of treatment for three months or extension of the dosing interval to 28 days for individuals receiving testosterone cypionate every 14 or 21 days. The discontinuation period of 3 months was calculated from the completion of the medication washout phase, defined as 15 days for individuals using testosterone cypionate and 90 days for those using testosterone undecanoate.

The variables evaluated were age at the time of diagnosis, presence of smoking and other addictions, parity, the presence or absence of amenorrhea, and laboratory results (including total T, hemoglobin [Hb], and Hct levels) at the time of diagnosis and 3 months after treatment. All laboratory data were collected from electronic medical records in the hospital laboratory system. Hct and Hb levels were measured using automated counting with the Siemens Advia 2120i device (Siemens Healthcare GmbH^®^, Munich, Germany). Total T levels were determined by radioimmunoassay, with readings performed on a Tri Carb Beta radiation counter (Perkin Elmer, Massachusetts, USA). The tests were conducted in the morning, between 7 and 9 AM.

Continuous variables are expressed as means and standard deviations, and qualitative variables are summarized as absolute and relative frequencies. The paired samples t-test was used to evaluate the effects of the interventions within the groups. A generalized linear model was used to verify the effect of the treatments on Hct and total T levels considering the changes between pre- and post-treatment measurements as outcome and the dose and therapeutic approach as covariates. Posttreatment variables were assessed 3 months after erythrocytosis was diagnosed and the therapeutic approach was initiated. Doses and interventions were compared by the least square means in the PROC GLM package of SAS software version 9.4 (SAS Institute Inc., Cary, NC, USA).

This study was conducted according to Declaration of Helsinki and approved by the Research Ethics Committee of the *Hospital das Clínicas da Faculdade de Medicina de Ribeirão Pre*to, University of São Paulo under Certificate of Submission for Ethical Appraisal, number 6.111.225 (*Certificado de Apresentação de Apreciação Ética*: 57815922.2.0000.5440). The need for participants’ consent was waived because this study analyzed data collected from medical records.

## Results

Among 181 trans men included, 49 (27.1%) were diagnosed with erythrocytosis and 22 (12.2%) had more than one erythrocytosis event. A total of 104 Hct tests that yielded levels of ≥50% were identified. Of these tests, 80 (76.9%) belonged to patients on testosterone cypionate treatment, 16 (15.4%) on testosterone undecanoate. The mean age of the participants was 28.0 ± 7.8 years; 10 (20.4%) were smokers, 20 (40.8%) had amenorrhea at the time of erythrocytosis diagnosis, and 8 (16.3%) had a previous pregnancy. At baseline, any participant presents previously history of thrombosis or had gone through hysterectomy or oophorectomy. Clinical and laboratory data are depicted in table 1. The mean and standard deviation of the Hct, Hb and total T levels at the time of diagnosis and 3 months later are presented in table 2.

**Table 1:** Clinical and laboratory data of transgender men diagnosed with erythrocytosis by group

Variables	Groups		
	Cip14	Cip28	Und90
Age (years)	26.7±7.3(56)	29.6±8.2(23)	29.9±8.4(16)
Testosterone level (ng/dL)	496.1±297.2(48)	275.0±205.0(20)	394.2±232.1(13)
Hemoglobin level (g/dL)	16.8±0.7(56)	17.0±0.7(23)	16.4±0.7(16)
Hematocrit level (%)	51.2±1.5(56)	51.5±1.4(23)	51.3±1.2(16)

Cip14, users of testosterone cypionate 200 mg/2 mL every 2 weeks; Cip28: users of testosterone cypionate 200 mg/2 mL monthly; Und90: users of testosterone undecanoate 1,000 mg/4 mL every 90 days. Data are presented as mean ± standard deviation (absolute sample size)

**Table 2:** Effects of the intervention on laboratory parameters by treatment

Variables	Baseline	3 months	p-value
Cip14 discontinuation			
Testosterone level (ng/mL)	553.16±256.20(31)	142.93±191.16(27)	<0.001
Hemoglobin level (g/dL)	16.86±0.65(35)	15.21±0.90(35)	<0.001
Hematocrit level (%)	51.57±1.67(35)	46.34±2.38(35)	<0.001
Cip14 dose spacing			
Testosterone level (ng/mL)	402.50±218.97(6)	155.50±180.59(8)	0.227
Hemoglobin level (g/dL)	16.71±0.52(9)	16.33±0.92(9)	0.171
Hematocrit level (%)	50.56±0.68(9)	49.11±3.00(9)	0.158
Cip 28 discontinuation			
Testosterone level (ng/mL)	269.00±191.91(11)	95.25±75.28(12)	0.009
Hemoglobin level (g/dL)	17.17±0.58(13)	15.06±1.01(12)	<0.001
Hematocrit level (%)	52.00±1.22(13)	44.83±3.16(12)	<0.001
Und90 discontinuation			
Testosterone level (ng/mL)	392.00 ± 145.42 (10)	227.96 ± 202.65 (5)	0.147
Hemoglobin level (g/dL)	16.47 ± 0.71 (13)	15.73 ± 0.88 (11)	0.008
Hematocrit level (%)	51.23 ± 1.01 (13)	48.65 ± 2.61 (11)	0.002

Cip14, users of testosterone cypionate 200 mg/2 mL every 2 weeks; Cip28: users of testosterone cypionate 200 mg/2 mL monthly; Und90: users of testosterone undecanoate 1,000 mg/4 mL every 90 days. Data are presented as mean ± standard deviation (absolute sample size)

In testosterone cypionate user (Cip14 and Cip28), as expected, T discontinuation reduced Hct, Hb and total T levels (Table 2). Discontinuation reduced more total T levels in Cip14 than in Cip28 (p=0.043), with no difference in reductions of Hct level (p=0.070; Table 3). In Und90, discontinuation resulted in decrease of Hct (p=0.002) and Hb (p=0.008) levels, with no difference in total T levels (p=0.147) (Table 2). When comparing the three groups, Cip14 and Cip28 exhibited greater reductions in the Hct level than Und90 did with discontinuation (Cip14 vs. Und90, p = 0.018; Cip28 vs. Und90, p<0.001) (Table 3).

**Table 3:** Variations during 3 months of interventions on hematocrit and serum testosterone levels according to testosterone cypionate and testosterone undecanoate use

Formulation	Discontinuation	Dose spacing
Hematocrit level (%)		
Cip14 group	−5.2±2.9(36)	−1.4±2.8(11)*†
Cip28 group	−7.1±2.9(13)	-
Und90 group	−2.7±2.2(13)*†	-
Testosterone level (ng/dL)		
Cip14 group	−442.4±344.8(36)	−232.0±413.2(11)
Cip28 group	−205.9±196.0(13)*	-
Und90 group	−103.8±106.7(13)*	-

n, absolute sample size; Cip14, users of testosterone cypionate 200 mg/2 mL every 2 weeks; Cip28, users of testosterone cypionate 200 mg/2 mL monthly; Und90: users of testosterone undecanoate 1,000 mg/4 mL every 90 days.

* p < 0.05 compared with discontinuation in the Cip14 group.

† p < 0.05 compared with discontinuation in the Cip28. Data are presented as mean ± standard deviation (absolute sample size)

In Cip14, dose spacing had no effect on decreasing Hct (p=0.158), Hb (p=0.171) and total T levels (p=0.227) (Table 2). Discontinuation was more effective in dropping Hct levels than dose spacing (p=0.001); and total T levels reduction was similar between the two treatments (p=0.135) (Table 3).

## Discussion

The objective of this study was to evaluate the effect of GAHT-T discontinuation and spacing on reducing the levels of Hct and total T in trans men. The literature indicates that 11% of trans men have erythrocytosis in the first year of GAHT-T,^(9)^ with testosterone undecanoate users being less likely to develop erythrocytosis than testosterone cypionate users.^(10)^ The estimation of a decrease in hematocrit values according to the formulation, associated with the maintenance of acceptable total T levels, which were the two variables evaluated in this study, can assist in decision-making regarding when to resume treatment for transmasculine individuals who need to suspend GAHT-T due to the development of erythrocytosis.

The efficacy and safety of T use and its effects on Hct in trans men has been demonstrated.^(1,4,6,8)^ A recent systematic review with meta-analysis showed that trans men undergoing GAHT-T had an increased Hb level of 1.48 g/dL (95% confidence interval, 1.17–1.78) and an increased Hct level of 4.39%, (95% CI, 3.52–5.26).^(4)^ However, that review evaluated only 312 trans men, without distinguishing between T formulations.

The discontinuation of treatment for 3 months in trans men with erythrocytosis is a therapeutic option based on the possibility that T-mediated hemoconcentration and erythrocyte senescence and phagocytosis in the spleen occurs approximately 120 days after T is produced.^(11,12)^ Developing more flexible management strategies for erythrocytosis is important to ensure the well-being of trans men because discontinuing GAHT-T can increase anxiety and gender dysphoria.^(13)^

Serum total T levels and erythrocytosis are positively correlated,^(11)^ with a shorter half-life justifying the more pronounced decrease in Hct levels in users of testosterone cypionate.^(9,14)^ Testosterone cypionate has a half-life of 6 to 8 days and fast decrease in the serum level of total T is expected after discontinuation;^(15)^ by contrast, testosterone undecanoate shows decreased levels approximately 10 to 12 weeks after discontinuation.^(16,17)^ In our study, testosterone undecanoate users were evaluated in 6 months after the last dose of T administration (3 months of discontinuation of GAHT-T), even so, total T levels at the point showed no difference from those at the moment of erythrocytosis diagnoses (baseline), with reduction in Hb and Hct levels. This effect may be able to mitigate side effects such as bleeding, gender dysphoria, and the regression of masculine characteristics in trans men.

Testosterone cypionate dose spacing from 14 to 28 days didn't improve the decrease of Hct levels and total T levels. Dose spacing to 28 days meant that total T levels were assessed at the lowest point due to its half-life of 6–8 days, justifying the finding that the levels of total T that were observed were similar to those observed with drug discontinuation.^(15)^ This observation should be evaluated with caution since our sample size is small in this group. Maintaining testosterone with fewer applications (monthly) may mitigate negative adverse effects associated with suspension. Furthermore, it is plausible to think that spacing out the doses could reduce the number of testosterone concentration peaks after administration, which, in the long term, could have a more prominent effect on Hct levels, although this conclusion was not observed in our results.

This study is the first to investigate the comparative effects of T discontinuation and dose spacing in trans men with erythrocytosis secondary to GAHT-T. It opens a horizon for the development of controlled protocols to determine the best strategy for treating erythrocytosis with the minimum number of adverse events. The laboratory data used in this study were analyzed by a single laboratory and were collected directly from system records to ensure homogeneous results

As a retrospective medical record review, this study had limitations, including bias of the clinical data resulting from the way they were described in the medical records. The patients confirmed the use of GAHT-T and self-reported its correct use, which may have resulted in information bias. Another limitation of this study was to consider only two timepoints for longitudinal assessment: the time at which erythrocytosis was diagnosed and 3 months after the purpose treatment, not being possible to identify a decay curve for each formulation. Another point is that despite the observed erythrocytosis rate in our service (27.1%), when analyzing the data in groups, the sample size became small, which may result in a higher likelihood of random errors.

## Conclusion

Discontinuation of testosterone undecanoate for 3 months in trans men undergoing GAHT-T who had developed erythrocytosis reduces hemoglobin and hematocrit levels without a significant reduction in testosterone levels. Dose spacing in fortnightly testosterone cypionate users was not effective to reduce hematocrit and hemoglobin. Further prospective studies with larger samples sizes are necessary to confirm the effects of testosterone formulations and to develop strategies to mitigate the testosterone discontinuation in this population.
